# Supplementary material for: Production of the Sesquiterpene Bisabolene From One‐ and Two‐Carbon Compounds in Engineered Methanosarcina acetivorans
Source: Microb Biotechnol. 2025 Feb 12;18(2):e70105. doi: 10.1111/1751-7915.70105 (PMC11816158; doi:10.1111/1751-7915.70105)
Supplement: Supplementary file 1 — Data S1. [file MBT2-18-e70105-s001.pdf]

Supporting Information

for

Production of the sesquiterpene bisabolene from one- and two-carbon compounds in engineered *Methanosarcina acetivorans*

Andrea Mentrup, Luca V. Scheitz, Theo Wallenfang and Michael Rother

Fakultät Biologie, Technische Universität Dresden, 01062 Dresden, Germany

This file includes:

Supplementary Tables S1-S4

Table S1: *M. acetivorans* strains used in this study.

Strain	Genotype	Reference
WWM73	Δ*hpt*::PmcrB-*tetR*-ΦC31-int-attP	(Guss et al., 2008)
MVA I	WWM73, Δ*ssu*::PmcrB(tetO1)-MM0870-MM0871-MM0335	This study
MVA II	WWM73, ΔMA3852::PmcrB(tetO3)-MM1526-MM1525-MM1524-MM1871	This study
MVA III	WWM73, ΔMA2965::PmcrB(tetO3)-MM1762-MM1763-MM1764	This study
MVA12	MVA I, ΔMA3852::PmcrB(tetO3)-MM1526-MM1525-MM1524-MM1871	This study
MVA13	MVA I, ΔMA2965::PmcrB(tetO3)-MM1762-MM1763(D_{31}N) -MM1764	This study
MVA23	MVA II, ΔMA2965::PmcrB(tetO3)-MM1762-MM1763(D_{31}N)-MM1764	This study
MVA231	MVA23, Δ*ssu*::PmcrB(tetO1)-MM0870-MM0871-MM0335	This study

Table S2: Plasmids used in this study[a].

Plasmid	Genotype and/or construction	Reference
pWM321	*M. acetivorans/E. coli* shuttle vector	(Metcalf et al., 1997)
pMR08	pWM321, PmcrB-*tetR*	(Oelgeschläger and Rother 2009)
pJK028a	ϕC31-*attB* vector (for genomic integration) with PmcrB(tetO3) promoter fusion to *uidA*	(Guss et al., 2008)
pMssu	Platform vector for chromosomal integration into the *ssu* locus (MA0063-MA0065)	(Sattler et al., 2024)
pMA2965	Platform vector for chromosomal integration into MA2965	(Sattler et al., 2024)
pMA3852	Platform vector for chromosomal integration into MA3852	(Sattler et al., 2024)
pUC57-BsaI-free	General cloning	Biocat GmbH, Heidelberg, Germany
pUC57_tetO1_Pt_APS	Codon optimized α-pinene synthase gene from *Pinus taeda* fused to PmcrB(tetO1) promoter	Biocat

pUC57_tetO1_Ms_LMS	Codon optimized limonene synthase gene of *Mentha spicata* fused to PmcrB(tetO1) promoter	Biocat
pUC57_tetO1_Sc_LIS	Codon optimized linalool synthase gene of *Streptomyces clavuligerus* fused to PmcrB(tetO1) promoter	Biocat
pUC57/tetO1_AgBIS_His_6	Codon optimized bisabolene synthase gene (with 3' His_6-tag encoded) of *Abies grandis* fused to PmcrB(tetO1) promoter	Biocat
pUC57_tetO4_MVA I	Artificial operon of the *M. mazei* genes MM_0335, MM_0870 and MM_0871 fused to PmcrB(tetO4) promoter	Biocat
pUC57_tetO3_MVA II	Artificial operon of the *M. mazei* genes MM_1526, MM_1525, MM_1524 and MM_1871 fused to PmcrB(tetO3) promoter	Biocat
pJK028a_MVA III	MM_1762, MM_1763 and MM_1764 from *M. mazei* (PCR amplified) fused to PmcrB(tetO3)	This study
pAPS	PmcrB(tetO1)-APS fusion (pUC57_tetO1_Pt_APS) moved to pMR08	This study
pLMS	PmcrB(tetO1)-LMS fusion (pUC57_tetO1_Ms_LMS) moved to pMR08	This study
pLIS	PmcrB(tetO1)-LIS fusion (pUC57_tetO1_Sc_LIS) moved to pMR08	This study
pAgBIS	PmcrB(tetO1)-AgBIS fusion PCR amplified, moved to pMR08	This study
pMssu_MVAI	PmcrB(tetO4)_MVA I fusion (pUC57_tetO4_MVA I)moved to pMssu	This study
pMA3852_MVA II	PmcrB(tetO3)_MVA II fusion pUC57_tetO3_MVA II) moved to pMA3852	This study
pMA2965_MVA III	PmcrB(tetO3)_MVA III fusion (pJK028a_MVA III) moved to pMA2965	This study
pMA2965_MVA III $D_{31}N$ MM1763	PmcrB(tetO3)_MVA III $D_{31}N$ MM1763 fusion (pJK028a_MVA III $D_{31}N$ MM1763) moved to pMA2965 (via PCR)	This study

a: sequences are available upon request.

Table S3: Oligonucleotides used in this study.

Oligonucleotide	Sequence (5'→3')
o_SphI_pmcrB_tetO1_for	AAAGCATGCTTCATTTATCGGAGAACAC
o_SpeI_HindIII_AgBIS_rev	AAAACTAGTAAGCTTAAAGCGGAAGCG
o_NdeI_Operon_MM1762_1764_for	TTTCATATGGTTTCATGTTCTGCGC
o_Operon_MM1762_1764_BamHI_rev	AAAGGATCCTTAAAGAGCGTTCC
o_NotI_pmcrB_tetO3_Operon_MM1762_64_for	TTTGCGGCCGCGCATGCTTCATTTATCG
o_pmcrB_tetO3_Operon_MM1762_64_NotI_rev	TTTGCGGCCGCCTTAAAGAGCGTTCC

Table S4: Summary of the *trans* genes used in this study.

Name	Encoded enzyme	ORF/accession number[a]	Organism	Comment	Reference
APS	α-pinene synthase	AF543530	*Pinus taeda*	Without plastid transport sequence, codon usage optimized	(Tashiro et al., 2016)
LMS	limonene synthase	L13459	*Mentha spicata*	Without plastid transport sequence, codon usage optimized	(Wu et al., 2019)
LIS	Linalool synthase	CP016560	*Streptomyces clavuligerus*	Codon usage optimized	(Nakano et al., 2011)
AgBIS	bisabolene synthase	AF006195	*Abies grandis*	Without plastid transport sequence, codon usage optimized	(Sebesta and Peebles 2020)
MVA I	acetoacetyl-CoA thiolase, HMG-CoA synthase, HMG-CoA reductase	MM_0870, MM_0871, MM_0335 (AE008384)	*Methanosarcina mazei*	Synthetic operon fused to PmcrB(tetO4)	(Yoshida et al., 2020)
MVA II	Pphosphomevalonate dehydratase, anhydromevalonate phosphate decarboxylase, prenylated flavin mononucleotide synthase	MM_1524, MM_1525, MM_1526, MM_1871 (AE008384)	*Methanosarcina mazei*	Synthetic operon fused to PmcrB(tetO3)	(Yoshida et al., 2020)

MVA III	mevalonate kinase, isopentenyl phosphate kinase, IPP:DMAPP isomerase	MM_1762, MM_1763, MM_1764 (AE008384)	*Methanosarcina mazei*	Operon fused to PmcrB(tetO3)	(Yoshida et al., 2020)

a: www.ncbi.nlm.nih.gov

References

Guss, A.M., Rother, M., Zhang, J.K., Kulkarni, G., and Metcalf, W.W. (2008) New methods for tightly regulated gene expression and highly efficient chromosomal integration of cloned genes for *Methanosarcina* species. *Archaea* **2**: 193-203.

Metcalf, W.W., Zhang, J.K., Apolinario, E., Sowers, K.R., and Wolfe, R.S. (1997) A genetic system for Archaea of the genus *Methanosarcina*: liposome-mediated transformation and construction of shuttle vectors. *Proceedings of the National Academy of Sciences of the United States of America* **94**: 2626-2631.

Nakano, C., Kim, H.-K., and Ohnishi, Y. (2011) Identification and characterization of the linalool/nerolidol synthase from *Streptomyces clavuligerus*. *ChemBioChem* **12**: 2403-2407.

Oelgeschläger, E., and Rother, M. (2009) *In vivo* role of three fused corrinoid/methyl transfer proteins in *Methanosarcina acetivorans*. *Mol Microbiol* **72**: 1260-1272.

Sattler, C., Richter, M., and Rother, M. (2024) Physiological barriers for glucose utilization in *Methanosarcina acetivorans*. *bioRxiv*: doi: 10.1101/2024.1108.1121.609014.

Sebesta, J., and Peebles, C.A.M. (2020) Improving heterologous protein expression in *Synechocystis* sp. PCC 6803 for alpha-bisabolene production. *Metabolic Engineering Communications* **10**: e00117.

Tashiro, M., Kiyota, H., Kawai-Noma, S., Saito, K., Ikeuchi, M., Iijima, Y., and Umeno, D. (2016) Bacterial production of pinene by a laboratory-evolved pinene synthase. *ACS Synth Biol* **5**: 1011-1020.

Wu, J., Cheng, S., Cao, J., Qiao, J., and Zhao, G.-R. (2019) Systematic optimization of limonene production in engineered *Escherichia coli*. *J Agric Food Chem* **67**: 7087-7097.

Yoshida, R., Yoshimura, T., and Hemmi, H. (2020) Reconstruction of the “archaeal" mevalonate pathway from the methanogenic archaeon *Methanosarcina mazei* in *Escherichia coli* cells. *Appl Environ Microbiol* **86**: e02889-02819.
